# Iodine-Based Electrolyte Chemistry Enabling Reversible Ca Metal Anodes

**DOI:** 10.1021/jacsau.5c01724

**Published:** 2026-02-02

**Authors:** Zhen Hou, Kai Liu, Rui Zhou, Chi Shing Tsang, Jiong Zhao, Junwu Zhu, Biao Zhang

**Affiliations:** † Key Laboratory for Soft Chemistry and Functional Materials, Ministry of Education, School of Chemistry and Chemical Engineering, 12436 Nanjing University of Science and Technology, Nanjing 210094, China; ‡ Department of Applied Physics & Research Institute for Smart Energy, 26680 The Hong Kong Polytechnic University, Hung Hom, Hong Kong 999077, China

**Keywords:** Ca metal, electrolyte, iodine, solid electrolyte interphase

## Abstract

Electrolyte chemistry is of paramount importance for tackling the challenge of irreversible Ca deposition/stripping caused by ionic-insulating solid electrolyte interphases (SEIs). Current research has been mainly concentrating on the boron center-based electrolytes despite their complex synthetic procedure and leaves aside others because of a virtually inhibited electrochemical response. Herein, we report a kind of iodine-based electrolytes comprising CaI_2_ salt paired with auxiliary iodides, in which the latter elevates the I^–^ concentration to reconfigure electrical double-layer structures of a low-solubility CaI_2_ electrolyte, thus accelerating Ca^2+^ desolvation and Ca^2+^ diffusion across SEI. Consequently, the optimized iodine electrolytes enable a high average Coulombic efficiency of 96.5% under 0.5 mAh cm^–2^ and a decent Ca reversibility at a large current density of 1.5 mA cm^–2^, showing competitive or even better performance than boron-based counterparts. As a proof of concept, full cells are demonstrated by coupling Ca metal anodes with an organic cathode, yielding an average output voltage of ∼2.1 V with outstanding stability for over 250 cycles. These findings expand the realm of Ca electrolyte chemistry, constituting a vital step in the development of efficient Ca systems.

## Graphical abstract

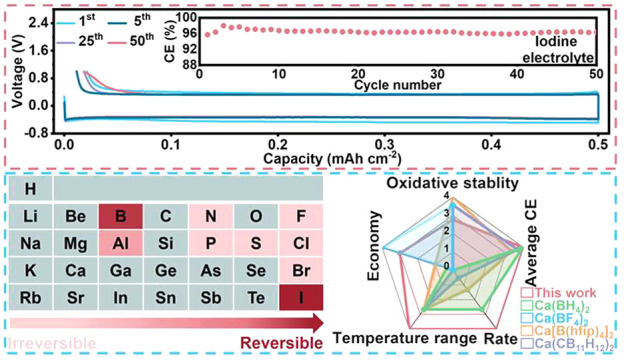

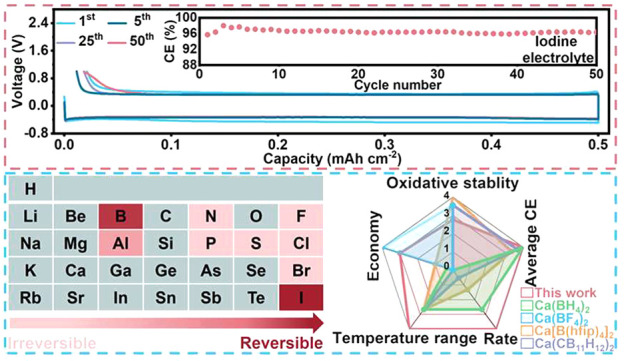

## Introduction

Research on divalent cation-based rechargeable batteries has surged in recent years.^1–4^ Multiple-electron redox chemistry brings about hope in breaking the energy density limitation of the monovalent counterpart, despite the challenges in designing appropriate host electrode materials.^5–8^ Compared to widely studied Zn and Mg, Ca batteries possess great advantages in achieving high energy density, attributed to the low redox potential of Ca/Ca^2+^ (i.e., −2.87 V vs the standard hydrogen electrode in contrast to −2.37 and −0.76 V for Mg/Mg^2+^ and Zn/Zn^2+^, respectively).^9–15^ The natural abundance of Ca elements will also benefit sustainable development.^16,17^ Nevertheless, early studies on Ca deposition/stripping in organic electrolytes suggest poor reversibility.^18,19^ Since the redox potential of Ca/Ca^2+^ goes beyond the stability window of most organic electrolytes, a solid electrolyte interphase (SEI) will be formed on the Ca metal surface, which consists of electrolyte decomposition products such as CaF_2_, CaO, and organic species. Unlike the ionically conductive SEI formed in Li-ion batteries, the one generated on Ca delivers a tremendous interfacial resistance that nearly blocks the Ca^2+^ transfer.^20–23^ The reason lies in the large Ca^2+^ diffusion barrier in the inorganic phases, where their monovalent counterparts like LiF and Li_2_O are favored for Li^+^ transfer.^24,25^

Reversible Ca deposition/stripping is essential for the development of not only rechargeable calcium metal batteries (RCMBs) but also other host electrodes, as Ca metal is an ideal counter/reference electrode. Extensive efforts have been devoted to electrolyte chemistry aimed at tailoring the SEI composition, particularly the inorganic component.^26–30^ Boron-center-based salts have received great attention, triggered by the reversible Ca deposition in the Ca­(BF_4_)_2_ electrolyte at elevated temperatures.^31^ Later efforts enable the operation at room temperature by developing new salts, such as calcium borohydride Ca­(BH_4_)_2_, calcium tetrakis­(hexafluoro­isopropyloxy)­borate Ca­[B­(hfip)_4_]_2_, and calcium monocarborane Ca­(CB_11_H_12_)_2_, and BF_4_-involved ionic liquid.^32–37^ Mechanism exploration indicates that calcium borate contributes to the improved kinetics at the interface. Other salts, including calcium fluorinated alkoxyaluminate Ca­[Al­(hfip)_4_]_2_ ^38^ and calcium tetrakis­(perfluoro-tert-butoxy) aluminate Ca­(TPFA)_2_,^39^ have also shown great potential for boosting Ca metal reversibility, due possibly to the similarity between B and Al.

Current Ca electrolyte chemistry is basically limited to boron/aluminum-based types despite their rigorous synthetic procedure, while reversible Ca deposition/stripping is nearly inhibited in others (as summarized in Table S1). To overcome this limitation, recent progress demonstrated that the CaI_2_ electrolyte was compatible with reversible Ca deposition/stripping.^40^ It was attributed to the unique SEI containing the ionically conductive CaI_2_ species, which possessed a significantly lower Ca^2+^ diffusion barrier than other calcium halides. However, a relatively large deposition/stripping overpotential persisted in pure CaI_2_ electrolyte owing to insufficient I^–^ concentration, stemming from the low solubility of CaI_2_ salt.

Herein, we increase the I^–^ concentration via simply introducing foreign iodides (e.g., LiI and KI), effectively boosting reaction kinetics to highly reduce voltage polarization. On the one hand, a higher I^–^ concentration weakens the interaction between Ca^2+^ and solvent molecules, promoting the Ca^2+^ desolvation process. On the other hand, the increased concentration results in an I^–^-rich electrical double layer (EDL), suppressing solvent decomposition to yield CaI_2_-rich/CaCO_3_-poor SEIs. Benefitting from the enhanced desolvation kinetics and Ca^2+^ diffusivity across SEI, Ca|Cu half cells deliver a high average Coulombic efficiency (CE) of 96.5% at 0.5 mA cm^–2^ in an optimal iodine electrolyte recipe, allowing the construction of Ca|3,4,9,10-perylene­tetracarboxylic diimide (PTCDI) full cells that stably run for 250 cycles. The Ca reversibility reported in this work surpasses that of most previously reported systems, demonstrating the fertile electrolyte chemistry beyond boron-based salts for Ca batteries.

## Results and Discussion

### Boosting Ca Reversibility in High I^–^ Concentration Electrolytes

The CaI_2_ electrolyte is compatible with Ca metal anodes, but its slow interfacial kinetics results in a huge deposition/stripping overpotential.^40^ The alteration of electrolyte concentration is the straightforward strategy to optimize the Ca^2+^ solvation sheath and EDL structure on the Ca surface, both of which play vital roles in dictating the interfacial kinetics.^2,41,42^ Therefore, we propose the addition of lithium iodide (LiI) and potassium iodide (KI) to the CaI_2_ electrolyte to increase the I^–^ concentration considering the poor solubility of CaI_2_. KI and LiI are employed as representative examples since both K^+^ and Li^+^ cations have a lower reduction potential than the Ca^2+^ cation for avoiding the introduction of foreign redox reactions (as discussed in Figure 2). Ca|Ca symmetric cells are employed to investigate their effects on kinetics. After adding KI (0.02 M) to the 0.02 M CaI_2_ electrolyte, the Ca deposition/stripping overpotential decreases to ∼0.65 V at 0.02 mA cm^–2^ (Figure 1a), an ∼4-fold reduction compared to that of the pure CaI_2_ electrolyte, indicating accelerated deposition/stripping kinetics. Besides, similar to CaI_2_/KI electrolytes, the 0.02 M CaI_2_/0.02 M LiI electrolyte shows comparable improvement, suggesting that enhanced Ca reversibility observed in these electrolyte systems is not primarily influenced by foreign cation types. Instead, this implies that the presence of more I^–^ anions may play a critical role in dictating Ca reversibility.

**Figure 1:**
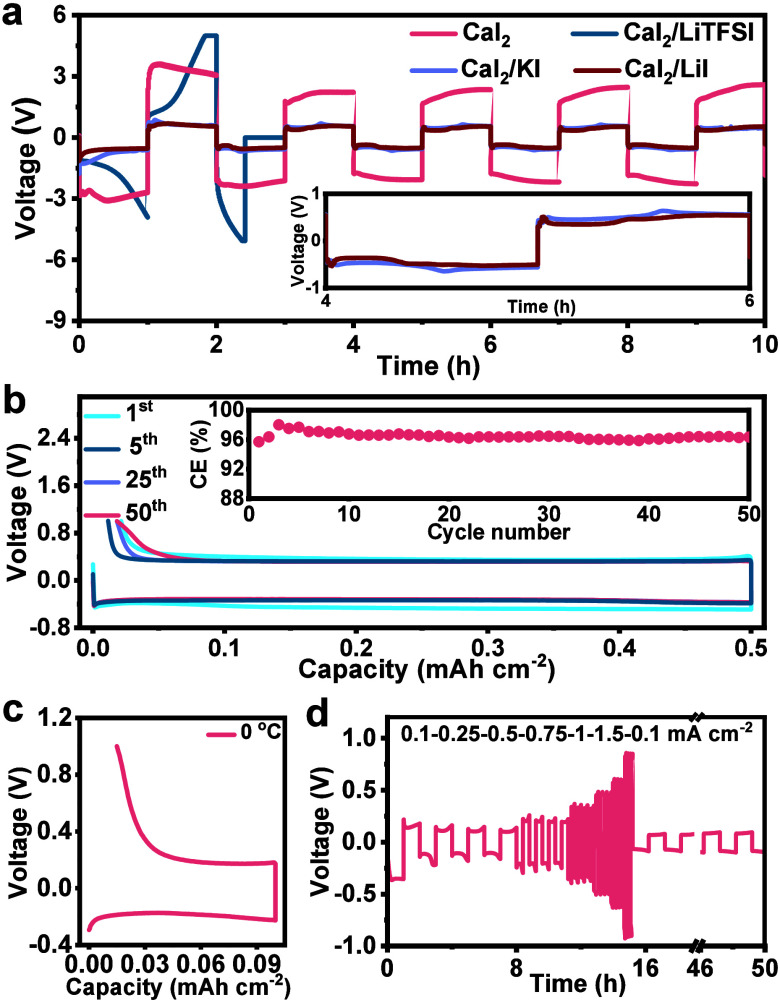
Ca reversibility in high I^–^ concentration electrolytes. (a) Cycling performance of Ca|Ca cells at 0.02 mA cm^–2^ in CaI_2_–, CaI_2_/LiTFSI–, CaI_2_/KI–, and CaI_2_/LiI–THF electrolytes. The additive concentration is 0.02 M. CE curves of Ca|Cu cells in CaI_2_/LiI–THF electrolyte at (b) 0.5 mA cm^–2^ under room temperature and (c) 0.1 mA cm^–2^ under 0 °C. The inset in (b) is the CE value. (d) Rate capability of the Ca|Ca cell in CaI_2_/LiI electrolyte. The LiI concentration in (b–d) is 0.2 M.

To validate this conjecture, we prepared a series of electrolytes utilizing an alternative Li salt or higher LiI concentrations. Note that we focus on the LiI system owing to its higher solubility in tetrahydrofuran (THF) solvent compared to that of KI. We first examine the role of the Li^+^ cation by using lithium bis­(trifluoromethanesulfonyl)­imide (LiTFSI) salt. A rapidly increased polarization voltage of over 5 V is observed by substituting LiTFSI for LiI (Figure 1a). This is because TFSI^–^ is highly susceptible to electrochemical reduction,^43,44^ forming a passivation layer rich in inert CaF_2_ species, which leads to a rapid increase in overpotential. This result suggests nearly irreversible Ca deposition/stripping and a negligible enhancement in the 0.02 M CaI_2_/0.02 M LiTFSI electrolyte. Namely, the strategy of incorporating Li salts alone cannot enhance Ca reversibility effectively in current system, demonstrating that the working mechanism of the CaI_2_/LiI electrolyte differs from that of previous studies using Li salts to improve the Ca deposition/stripping overpotential.^37,45^ We next explore the impact of I^–^ concentration by controlling the LiI amount under 0.02 M CaI_2_ electrolytes. As expected, increasing the LiI concentration from 0.02 to 0.1 M results in a decreased polarization voltage to ∼0.15 V (Figure S1), and this value further reduces to ∼0.03 V under a higher LiI concentration of 0.2 or 0.4 M. Therefore, we will mainly center on the 0.02 M CaI_2_/0.2 M LiI (named CaI_2_/LiI hereinafter for clarity) electrolyte system to investigate the working mechanism of foreign iodides because 0.2 M LiI concentration is sufficient to enable optimal Ca reversibility.

To further demonstrate the effectiveness of the CaI_2_/LiI electrolyte, the Coulombic efficiency (CE), another critical parameter to assess Ca reversibility, is measured in Ca|Cu cells where Cu serves as the working electrode. The CaI_2_/LiI electrolyte enables a high average CE of 96.5% under a current density of 0.5 mA cm^–2^ and a cycling capacity of 0.5 mAh cm^–2^ for over 50 cycles (Figure 1b and Figure S2). The cell sustains an acceptable CE even at a low temperature of 0 °C (Figure 1c). In addition, the CaI_2_/LiI electrolyte endows an encouraging rate capability, maintaining decent reversibility at a high current density of up to 1.5 mA cm^–2^ (Figure 1d). The performance surpasses that of most of the previously reported Ca metal anodes in terms of CE and rate capability (Table S1).

### Verifying the Ca/Ca^2+^ Redox Reaction

As both Li^+^ and Ca^2+^ are present in the electrolyte, we verify that the above electrochemical behavior is nested in the Ca/Ca^2+^ redox reaction. Theoretically, the standard redox overpotential of Ca/Ca^2+^ is 0.17 V higher than that of Li/Li^+^, despite the lower concentration of the former, which is apt to avoid Li metal deposition during the Ca^2+^ electro-reduction process (detailed calculation in Note S1).^46^ To determine the threshold value of the Ca^2+^ concentration for initiating Ca deposition, we examine the deposit species using Ca|Cu cells under different deposition capacities in pure LiI electrolyte. The Ca electrode is oxidized to gradually increase the Ca^2+^ concentration in the electrolyte. It is found that a 0.007 M CaI_2_ concentration is sufficient to support Ca deposition from the electrolyte (Figure S3). These results confirm the preferred Ca^2+^ electro-reduction in CaI_2_/LiI electrolyte, even under an extremely low concentration of CaI_2_.

To exhaustively exclude the artifact from the Li/Li^+^ redox reaction in the CaI_2_/LiI electrolyte, we conduct a collection of spectroscopy analyses and theoretical calculations. The samples are prepared by depositing metal ions on Cu current collectors using Ca|Cu cells. Scanning electron microscopy (SEM) images show that a few deposits are formed under 0.2 mAh cm^–2^ (Figure 2a). As the deposition capacities increase to 0.5 and 1 mAh cm^–2^, the deposits gradually cover the Cu current collector (Figure 2b and c). The corresponding energy dispersive spectroscopy (EDS) elemental mappings demonstrate that Ca element signals are well overlapped with the deposits under all deposition capacities (Figure 2d and e and Figure S4). Meanwhile, the EDS spectrum also manifests intense Ca characteristic signals (Figure 2f). These observations indicate that the Ca species is the dominant component in the deposits. Given the insensitive response of the Li characteristic signal in the EDS measurement, we collect X-ray diffraction (XRD) patterns of the deposits. The diffraction peaks correspond to Ca metal as the sole phase without Li metal (Figure 2g), providing direct evidence that the deposits are metallic Ca. Moreover, X-ray photoelectron spectroscopy (XPS) analysis is carried out to inspect whether amorphous Li metal exists in the deposits. There is an absence of any Li 1s signal on the surface (Figure 2h). Even after Ar ion sputtering for 1500 s, the Li characteristic signal is undetected, unambiguously proving that Li^+^ is not reduced from the electrolyte. This phenomenon is explained by calculating the cations’ lowest unoccupied molecular orbital (LUMO) in the CaI_2_/LiI electrolyte. It is observed that the LUMO of Li^+^ is higher than that of Ca^2+^ by ∼1 eV, supporting the Ca^2+^ reduction prior to Li^+^ during electrodeposition in THF solvent (Figure 2i and Figure S5).^47^ Therefore, these cross-validated results thoroughly eradicate the possibility of Li^+^ electro-reduction.

**Figure 2:**
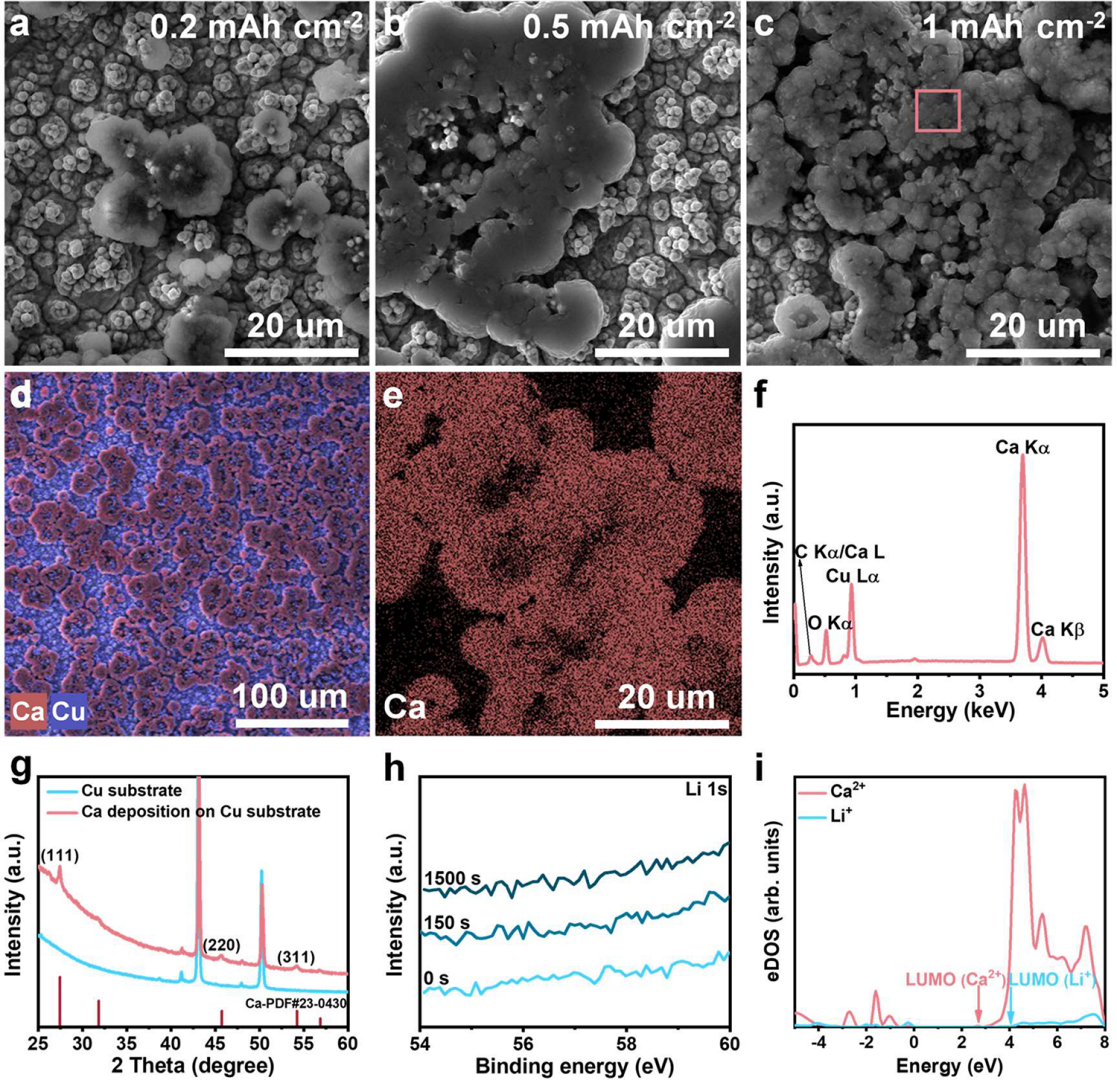
Characterization of the deposits on the Cu current collector at 0.5 mA cm^–2^. SEM images of Ca deposition under (a) 0.2 mAh cm^–2^, (b) 0.5 mAh cm^–2^, and (c) 1 mAh cm^–2^. (d, e) Corresponding EDS elemental mappings under 1 mAh cm^–2^. (f) EDS spectrum within the pink squared area in (c). (g) XRD patterns of a Cu current collector with and without 1 mAh cm^–2^ Ca deposition. (h) Li 1s XPS profiles of a Cu current collector with 1 mAh cm^–2^ Ca deposition. (i) Projected electronic density of states (eDOS) of Ca^2+^ and Li^+^ in CaI_2_/LiI electrolyte with a Perdew–Burke–Ernzerhof level of theory. Note that the Fermi level is shifted to 0 eV.

### Unraveling the Working Mechanism in High I^–^ Concentration Electrolytes

The above results demonstrate the enhanced Ca deposition/stripping behavior under CaI_2_/LiI electrolytes. We next focus on the Ca^2+^ desolvation process and diffusion across SEI, the two major processes governing deposition/stripping reversibility,^48^ to disclose the roots of unique I^–^-rich electrolytes. Electrochemical impedance spectroscopy (EIS) measurements are performed using cycled Ca|Ca cells in CaI_2_ and CaI_2_/LiI electrolyte systems (Figure 3a). Their Nyquist plots are fitted by an equivalent circuit consisting of bulk resistance (R_b_), SEI resistance (R_SEI_), and charge transfer resistance (R_ct_) (Figure S6). Under this circumstance, R_ct_ mainly refers to the Ca^2+^ desolvation barrier.^49^ As shown in Figure 3b, LiI addition leads to optimized Ca^2+^ desolvation kinetics, as proven by the decreased R_ct_ from ∼40.2 to ∼3.5 kΩ. Most importantly, a higher I^–^ concentration reduces R_SEI_ by about 2 orders of magnitude (i.e., from ∼398.6 kΩ in CaI_2_ to ∼4.4 kΩ in CaI_2_/LiI electrolytes, confirming a significantly boosted Ca^2+^ diffusion kinetics in SEI. To reveal their SEI discrepancy, we carry out depth-profiling XPS and transmission electron microscopy (TEM). A high I^–^ concentration maintains an SEI species similar to that of pure CaI_2_ electrolyte, containing C–C, C–O, Ca–O, and Ca–I bonds (Figure 3c and Figures S7 and S8). However, the CO_3_ bond associated mainly with the CaCO_3_ species almost disappears, as observed in the deconvoluted C 1s spectra. These observations are further confirmed by TEM results (Figure 3d and e). All SEIs consist of an amorphous polymer matrix with minor crystal phases embedded. Specifically, they share CaO and CaI_2_ crystals, while CaCO_3_ are observed only in pure CaI_2_ electrolyte SEIs. Note that CaCO_3_ has a diffusion barrier of ∼1.2 eV that is much higher than 0.4 eV for CaI_2_ (Figure S9), making it unfavorable for Ca metal anodes. In addition, with a higher I^–^ concentration in the electrolyte, the I content in SEI is almost doubled before and after sputtering (Figure S10a), demonstrating the increased CaI_2_ species in SEI under the CaI_2_/LiI electrolyte. This is similar to the previous alkali metal anodes’ studies where high-concentration electrolytes are preferred to induce the salt-derived SEIs.^50^

**Figure 3:**
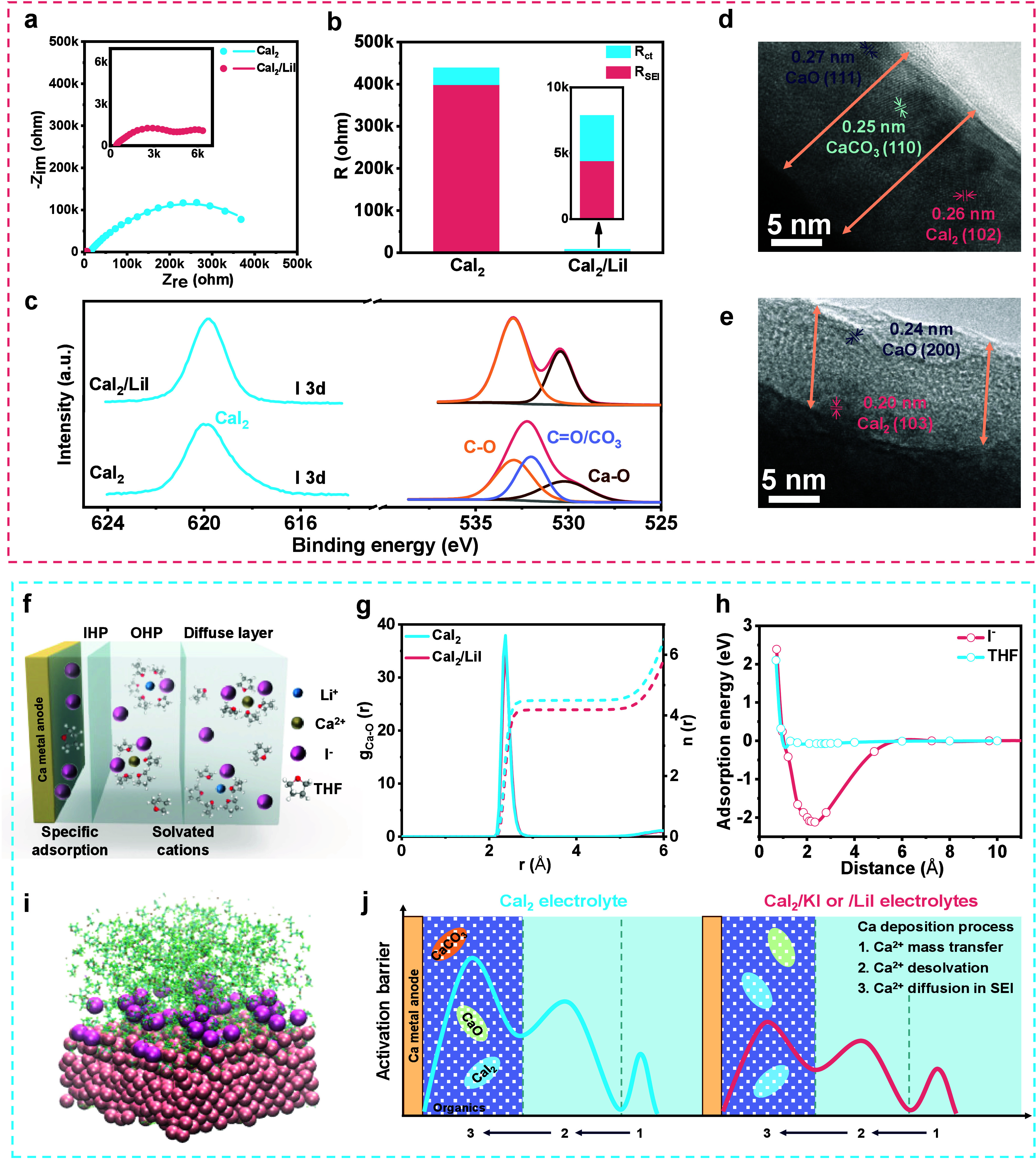
Effects of I^–^ concentration on electrical double-layer structures. (a) EIS Nyquist plots where dots and lines respectively represent measured and fitting data and (b) the corresponding R_SEI_ and R_ct_ fitting results of cycled Ca|Ca cells. The insets in (a) and (b) are the enlarged Nyquist plot and resistance values of the CaI_2_/LiI electrolyte, respectively. (c) O 1s and I 3d spectra of the SEIs formed on Ca metal anodes after Ar ion sputtering for 150 s. TEM images of the SEIs formed in (d) CaI_2_ and (e) CaI_2_/LiI electrolytes. (f) Schematic description of EDL structure in CaI_2_/LiI electrolyte. (g) Ca–O_THF_ radial distribution functions and integrated coordination number in CaI_2_ and CaI_2_/LiI electrolytes from MD simulations. (h) Energies of Ca–X with different X-to-surface distances, where X = THF or I^–^. (i) Snapshots of MD simulations of the IHP in the CaI_2_/LiI–THF electrolyte. (j) Illustrations of activation barriers in CaI_2_ and CaI_2_/KI or/LiI electrolytes during the Ca^2+^ desolvation process and diffusion process across SEIs.

Knowing the positive roles of a high I^–^ concentration in promoting the desolvation process and optimizing SEI species, we aim to explore the working mechanism. To disclose the distinction under different I^–^ concentrations, we focus on these electrolytes’ EDL structures that dominate the SEIs and kinetics at the interface.^41,42^ The EDL structure consists of the inner Helmholtz plane (IHP) and the outer Helmholtz plane (OHP). Specific adsorption of anions/molecules is located in the former, and the latter includes the solvated cations (Figure 3f).^51^ Solvation structures of CaI_2_ and CaI_2_/LiI electrolytes are simulated to understand the faster desolvation process in the latter. At a higher I^–^ concentration in CaI_2_/LiI electrolyte, more I^–^ participated in the Ca^2+^ solvation sheath (Figure S11), weakening the interaction between Ca^2+^ and THF molecules. This gives rise to the decreased average number of O_THF_ in the first solvation sheath of Ca^2+^ from 4.49 to 4.18 (Figure 3g), thus promoting the desolvation kinetics,^37,52^ consistent with the reduced R_ct_ as discussed in Figure 3a. Adsorption conditions on the Ca surface in the IHP are assessed via adsorption energy calculations and MD simulations. It is observed that there is a strong adsorption tendency of I^–^ on the Ca surface owing to its higher adsorption energy of ∼−2.8 eV, compared with ∼−0.1 eV for THF (Figure 3h). Despite the significantly strong specific adsorption of I^–^, IHP still contains rich THF molecules under a low I^–^ concentration. The higher I^–^ concentration because of LiI addition is expected to rearrange I^–^ and THF within the IHP. This is proven by the alternating current voltammetry showing the positively shifted potential of zero charge in the CaI_2_/LiI electrolyte (Figure S12a), which indicates that more I^–^ anions are absorbed in the IHP.^53,54^ The formation of an I^–^-occupied IHP is further confirmed by the snapshots from MD simulations. As shown in Figure 3i and Figure S12b, a higher I^–^ coverage is observed in CaI_2_/LiI electrolytes. Therefore, a high I^–^ concentration gives rise to more I^–^ and less THF in both IHP and OHP, which leads to the suppressed solvent decomposition that generates the CaCO_3_ component^55,56^ and the increased CaI_2_ species in SEI, both of which benefit Ca^2+^ diffusion.

Based on these observations, we depict the working mechanism of high I^–^ concentration electrolytes in Figure 3j. The pure CaI_2_ electrolyte suffers from sluggish reaction kinetics owing to its derived SEI containing insufficient ionically conductive CaI_2_ species. The foreign iodide additions (e.g., KI and LiI) increase the I^–^ concentration in electrolytes, promoting I^–^-rich solvation structure and CaI_2_-rich/CaCO_3_-poor SEI for the enhanced desolvation kinetics and Ca^2+^ diffusivity across SEI.

### Constructing Full Cells

Last, we assess the practical application of the CaI_2_/LiI electrolyte by pairing Ca metal anodes with a PTCDI organic cathode (Figure S13a).^30,57^ Thanks to the decent electrochemical window of over 3.1 V (Figure S13b), the electrolyte can support the reversible operation of Ca|PTCDI full cells between 1.0 and 2.8 V. Specifically, full cells are discharged/charged at 100 mA g^–1^ for over 250 cycles, delivering an average discharge voltage of ∼2.1 V with a reversible capacity of ∼81 mAh g^–1^ (Figure 4a). Furthermore, they maintain decent stability under 0 °C. It is worth mentioning that low-temperature Ca metal batteries have rarely been achieved due to sluggish reaction kinetics. The full cells also possess a good rate capability where a specific capacity of ∼47 mAh g^–1^ is maintained at a large specific current of 0.8 A g^–1^ (corresponding to an ∼10 C rate) (Figure 4b). EDS elemental mappings prove the Ca^2+^ involvement in the cathodes’ redox reactions (Figure S14a–h). Besides, an inductively coupled plasma–mass spectrometry (ICP–MS) measurement of a discharged PTCDI electrode shows that around 98% capacity comes from Ca^2+^ insertion while the rest is contributed by Li^+^ insertion (Figure S14i). This is similar to previous studies on organic cathodes under a hybrid Mg^2+^/Li^+^ electrolyte, where Li^+^ hardly takes part in the storage reactions.^58^ These results prove the feasibility of iodine-based electrolytes for implementing reversible Ca metal batteries.

**Figure 4:**
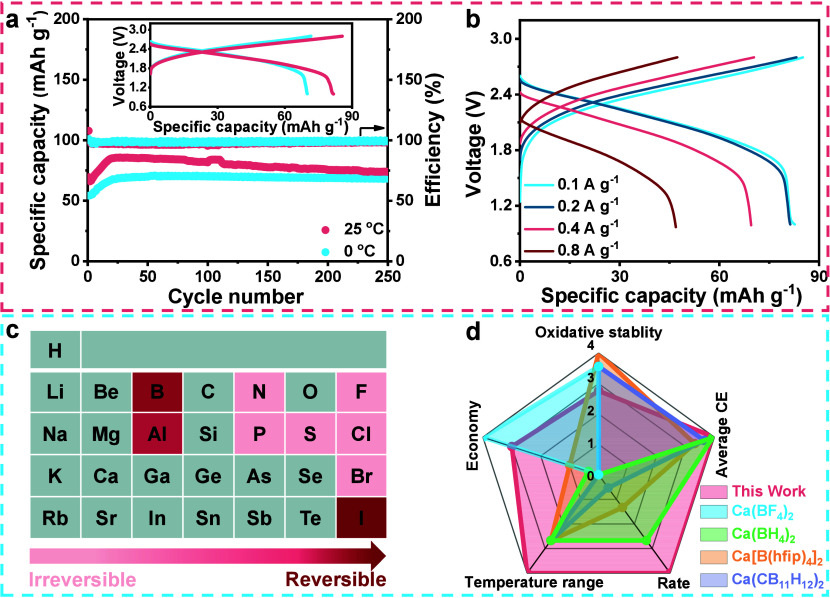
Ca|PTCDI full cell demonstration and overall evaluation of the iodine-based electrolytes. (a) Cyclic stability at 100 mA g^–1^ under 0 and 25 °C. The inset shows the detailed voltage curves. (b) Rate performance at 25 °C. (c) Modified periodic table demonstrating the correlation between different element-based electrolytes and Ca deposition/stripping reversibility. (d) Radar plot comparing our work and the reported boron-based electrolytes^31–36^ in terms of five angles. A rating of 0 represents poor oxidative stability, high processing cost, unsatisfactory temperature adaptiveness, rate capability, and CE performance, while 4 stands for the ideal properties among these electrolytes.

## Conclusions

This work proposes a class of iodine-based electrolytes (e.g., CaI_2_/KI or/LiI) for reversible Ca metal anodes, showing competitive or even better performance than the widely explored boron-based counterpart, both of which significantly outperform others such as P- (Ca­(PF_6_)_2_)- and S-center (Ca­(CF_3_SO_3_)_2_)-based electrolytes (Figure 4c). The addition of KI or LiI increases the I^–^ concentration in the electrolyte, regulating EDL structure to yield an I^–^-rich solvation sheath in OHP and I^–^-occupied IHP. Such reconstruction concurrently accelerates Ca^2+^ desolvation and rapid Ca^2+^ diffusion within SEI. Consequently, the optimal iodine-based electrolytes enable an attractive CE of 96.5% on average under 0.5 mA cm^–2^ and maintain reversible Ca deposition/stripping at a high current density of 1.5 mA cm^–2^. Their moderate oxidative stability allows the construction of a Ca-based full cell using cathodes with a suitable voltage. As a proof of concept, a 2.1 V class Ca|PTCDI full cell is demonstrated and reversibly runs for 250 cycles under 0 and 25 °C. We comprehensively evaluate five criteria among the reported electrolytes and our work (Figure 4d). Overall, Ca systems demonstrate the preferable CE, rate capability, temperature adaptiveness, and economy under iodine-based electrolytes, showing great potential for developing Ca metal batteries.

Notably, though comprehensive analyses have proven that Li^+^ is not reduced and our working mechanism of incorporating LiI salt differs from previous reports using multimetal ions, the low reserve of elemental Li would reduce the sustainability of Ca batteries. Meanwhile, the CE remains below 99%, a threshold that has not yet been attained in current research. Such a gap is probably due to insufficient inorganic species within the SEI layer under a low salt concentration condition, which limits its ability to effectively suppress side reactions between the electrolyte and Ca metal anode. Future research necessitates the exploitation of more appropriate candidates to improve the I^–^ concentration without resorting to LiI to increase the sustainability, such as employing higher solvating solvents to promote CaI_2_ dissociation, therefore fostering a denser inorganic-rich SEI layer that better protects the Ca metal anode. We hope that these findings will advance the development of Ca electrolytes and their associated battery technologies.

## Supplementary Material



## Notes

Experimental section; additional theoretical calculation details, electrochemical performance, spectral characterization, and SEM images (PDF)
