# Supplementary material for: Iodine-Based Electrolyte Chemistry Enabling Reversible Ca Metal Anodes
Source: JACS Au. 2026 Feb 2;6(2):1382–9. doi: 10.1021/jacsau.5c01724 (PMC12933320; doi:10.1021/jacsau.5c01724)
Supplement: Supplementary file 1 [file au5c01724_si_001.pdf]

**Supporting Information**

# Iodine-based electrolyte chemistry enabling reversible Ca metal anodes

Zhen Hou[a,b], Kai Liu[a], Rui Zhou[b], Chi Shing Tsang[b], Jiong Zhao[b], Junwu Zhu[a,*] and Biao Zhang[b,*].

[a]Key Laboratory for Soft Chemistry and Functional Materials, Ministry of Education, School of Chemistry and Chemical Engineering, Nanjing University of Science and Technology, Nanjing 210094, China.

[b]Department of Applied Physics & Research Institute for Smart Energy, The Hong Kong Polytechnic University, Hung Hom, Hong Kong 999077, China.

## Experimental Section

### Preparation of Ca electrolytes

lithium bis(trifluoromethanesulfonyl)imide (LiTFSI, 99.90%) were purchased from DoDoChem. Lithium iodide (LiI, 99.99%) and potassium iodide (KI, 99.99%) were purchased from Aladdin. Calcium iodide ($CaI_2$, 99.00%) and tetrahydrofuran (THF, 99.00%) were purchased from Sigma-Aldrich. A collection of electrolytes were prepared, including $CaI_2$ electrolyte (0.02 M $CaI_2$ in THF), $CaI_2$/LiTFSI electrolyte (0.02 M $CaI_2$ and 0.02 M LiTFSI in THF), $CaI_2$/KI electrolyte (0.02 M $CaI_2$ and saturated KI in THF) and $CaI_2$/LiI electrolytes (0.02 M $CaI_2$ and 0.02 M, 0.1 M, 0.2 M, or 0.4 M LiI in THF).

### Characterizations

Scanning electron microscope (SEM) images were conducted on Tescan VEGA3. Samples were sealed in an argon-filled air-tight container and quickly transferred into the SEM chamber. Transmission electron microscopy (TEM) images were collected using JEOL JEM-2100F. Ca deposition/stripping process was applied to TEM grids to generate solid electrolyte interphases. X-ray diffraction (XRD) was carried out on an X-ray diffractometer (Rigaku SmartLab) with Cu Ka radiation. The samples were sealed with Kapton tape to avoid oxidation. X-ray photoelectron spectroscopy (XPS) was tested using an X-ray photoelectron spectrometer (Nexsa) with an Al Ka X-ray line. Samples were transferred into the chamber through an argon-filled transfer container without exposure to air.

### Electrochemical measurements

Ca|Ca symmetric cells were assembled using CR2032 coin cell configuration where Ca metal serves as the working and counter/reference electrodes. Ca|Cu cells consist of a Cu working electrode and a Ca counter/reference electrode. Each cell contains Ca anode (purchased from Beijing Zhongke Yannuo New Material Technology Co., Ltd) with a diameter of 10 mm, one piece of glass fiber and 80 μL electrolyte. All cells were prepared in an argon-filled glovebox. Cyclic stability and Coulombic efficiency were tested on the Neware battery testing system. Linear sweep voltammetry (LSV) and electrochemical impedance spectroscopy (EIS) were collected on the BioLogic electrochemical workstation (VSP). EIS was carried out from $10^5$ Hz and $10^{-1}$ Hz (potential amplitude of 5 mV). Full cells were assembled by coupling Ca metal anodes with 3,4,9,10-perylenetetracarboxylic diimide (PTCDI) cathodes. PTCDI slurry consisting of 50 wt% PTCDI (95.00%, Aladdin), 20 wt% Super P, 30 wt% polyvinylidene difluoride was cast into watch glass to obtain free-standing PTCDI cathodes.

**Computational methods**

Density functional theory calculations on migration barriers were carried out using VASP6.3.[1] Generalized gradient approximation (GGA) with the Perdew-Burke-Ernzerhof (PBE) functional was used with a planewave cutoff of 520 eV. A climbing image nudged elastic band was used to compute the migration barrier of bulk phases. For these calculations, supercells were created with one $Ca^{2+}$ vacancy introduced.[2] Additional background charge was added to maintain the neutrality of the simulation cell and valances of anions. Five images were used with a force convergence criterion

of 0.01 eV/Å. Adsorption energies were calculated via a slab model where the adsorbates were fixed at different distances to the Ca surface and the energies were computed. In particular, the coordinates of I and O along the z-axis (the vacuum direction) and all coordinates of the lowest Ca layer were fixed. All the other coordinates were relaxed till the energy change was smaller than $10^{-3}$ eV. Van der Waals correction was added to correct interactions between THF and Ca surfaces.[3]

Molecular dynamics simulations were conducted using GROMACS version 5.1.2.[4] All-atom optimized potentials for liquid simulations (OPLS-AA) force field were employed to describe the interatomic interactions.[5] Partial charges were derived from the restrained electrostatic potential charge obtained via density functional theory calculations at the B3LYP/6-31G* level. Initial configurations for two electrolytes, i.e., 0.02 M $CaI_2$-THF and 0.02 M $CaI_2$/0.2 M LiI-THF, were generated using packmol, with each system containing over 8000 atoms.[6] Following initial energy minimization, production runs of 10 ns long NPT trajectories were performed, followed by a second 10 ns NPT simulation. Molecular dynamics simulations of the inner Helmholtz plane were carried out using LAMMPS with an in-house developed machine learning forcefield trained on GGA-PBE level DFT data.[7-10] The models were constructed with slightly higher concentrations compared with experimental value to minimize the computational cost. The minimum number of atoms was 3000. The simulation timestep was set to 1 fs and the total simulation time was 100 ps for each system. An NPT thermostat was used to control the pressure to ambient pressure and the temperature to 300 K.

**Note S1**

Estimation of the thermodynamic deposition potentials of $Li^+$ and $Ca^{2+}$ is carried out using the Nernst equation:

$$E_{red} = E_{red}^0 - \frac{RT}{zF} Q$$

where $E_{red}$ and $E_{red}^0$ are the cell reduction potential and the standard cell reduction potential, respectively. $R$ is the universal gas constant. $T$ is the temperature in kelvins. $z$ is the number of electrons transferred in half-reaction. $F$ is the Faraday constant. $Q$ is the reaction quotient. By taking room temperature for T and 1 for the activity coefficients ( $\gamma_{Li^+}$ or $\gamma_{Ca^{2+}}$), the formula can be rewritten to account for the deviation of

$$\begin{aligned} \Delta E_{dep_{Li}} &= E_{dep_{Li}} - E_{red_{Li}}^0 \\ &= -\frac{RT}{zF} Q \\ &= -\frac{RT}{zF} log\left(\frac{1}{\gamma_{Li^+} c_{Li^+}}\right) \\ &= -0.0592\, V\, log\left(\frac{1}{c_{Li^+}}\right) \\ &= -0.041\, V \end{aligned}$$

and

$$\begin{aligned} \Delta E_{dep_{Ca}} &= E_{dep_{Ca}} - E_{red_{Ca}}^0 \\ &= -\frac{RT}{zF} Q \\ &= -\frac{RT}{zF} log\left(\frac{1}{\gamma_{Ca^{2+}} c_{Ca^{2+}}}\right) \\ &= -0.0291 V\, log\left(\frac{1}{c_{Ca^{2+}}}\right) \\ &= -0.050\, V \end{aligned}$$

where $C_{Li^+}$ and $C_{Ca^{2+}}$ are the concentrations of $Li^+$ and $Ca^{2+}$ in 0.02 M $CaI_2$/0.2 M LiI

electrolyte.

Therefore, the relative difference in deposition potential deviation caused by different $Li^{+}$ and $Ca^{2+}$ concentrations is

$$\Delta\Delta E_{dep} = \Delta E_{dep_{Li}} - \Delta E_{dep_{Ca}} = +0.009\ \mathrm{V}$$

Considering $E^{0}_{red_{Ca}}$ and $E^{0}_{red_{Li}}$ have a difference of +0.17 V >> $\Delta\Delta E_{dep}$, the order of electrodeposition will not change by altering the solution concentration.

**Table S1.** Summary of electrolyte formulation, Ca deposition/stripping reversibility and full cell performance in the previous reports. "None" means the data is not provided in the references.

| Electrolyte type | Electrolyte formulation | Average CE for cycle numbers | Maximum current density | Cycle numbers of full cell | Testing temperature | Ref. |
|---|---|---|---|---|---|---|
| Iodine-based | $CaI_2$/LiI in THF | 96.5% for 50 cycles | 1.5 mA $cm^{-2}$ | Ca\|PTCDI: 250 cycles | 0 and 25°C | This work |
| Boron/aluminum -based | $Ca(BF_4)_2$ in EC/PC | Low CE for 30 cycles | None | None | ≥75°C | 11 |
| | $Ca(BH_4)_2$ in THF | ~95.0% for 50 cycles | 1.0 mA $cm^{-2}$ | None | 25°C | 12 |
| | $Ca(BH_4)_2$ in $[Ca(BH_4)_2]_{0.05}[N_{07}TFSI]_{0.95}$ | ~37.0% for 10 cycles | None | Ca\|$V_2O_5$: 4 cycles | 25°C | 13 |
| | $Ca[B(hfip)_4]_2$ in DME | ~80.0% for 20 cycles | 0.5 mA $cm^{-2}$ | None | 25°C | 14, 15 |
| | $Ca(CB_{11}H_{12})_2$ in DME/THF | ~88.0% for 30 cycles | None | Ca\|S: 1 cycle | 25°C | 16 |
| | $Ca(TFSI)_2$ in EC/PC with $BF_3$·DE additive | ~30.0% for 25 cycles | None | None | 100°C | 17 |
| | $Ca[Al(hfip)_4]_2$ in DME | ~65% for 50 cycles | None | Ca\|NP: 9 cycles | 25°C | 18 |
| | $Ca(TPFA)_2$ in DME | ~55.0% for 1 cycle | None | Ca\|CuS: 4 cycles | 25°C | 19 |
| Others | $Ca(TFSI)_2$ in EC/PC or THF | Irreversible | | | | 11 |
| | $Ca(ClO_4)_2$ or $Ca(BF_4)_2$ in PC, ACN, THF and γ-BL | | | | | 20 |
| | $Ca(AlCl_4)_2$ in $SOCl_2$ | | | | | 21 |

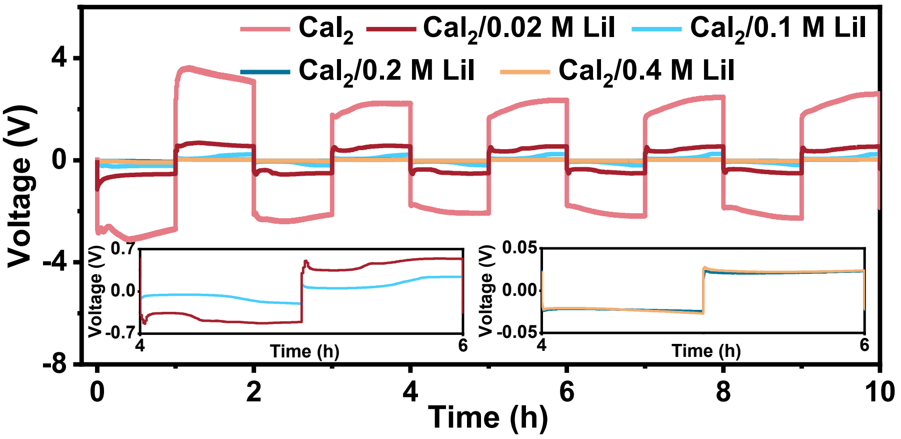


**Figure S1.** Cycling performance of Ca|Ca cells at 0.02 mA $cm^{-2}$ in 0.02 M $CaI_2$- and 0.02 M $CaI_2$/various concentrations LiI-THF electrolytes.

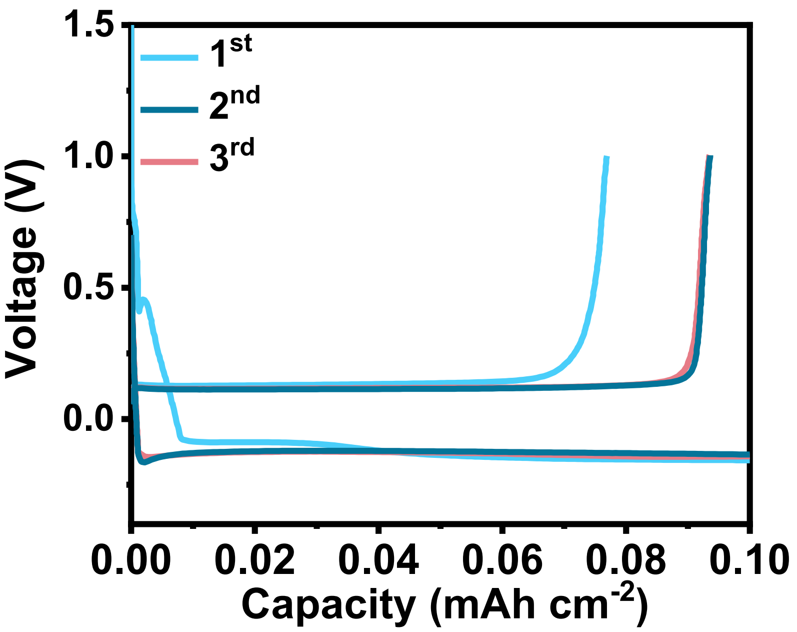


**Figure S2.** Pre-activated cycles of Ca|Cu cell in $CaI_2$/LiI electrolyte at 0.1 mA $cm^{-2}$ under room temperature. Before testing the Ca|Cu cell at 0.5 mA $cm^{-2}$ for 0.5 mAh $cm^{-2}$, the cell is pre-activated at 0.1 mA $cm^{-2}$ for 3 cycles.

Pre-activation cycles are conducted to remove impurities (e.g., native oxides) of Cu and Ca current collector, enhancing the stability of SEI.

In addition, the overpotential appears slightly higher during the initial cycling (**Figure 1d**), primarily attributed to interfacial evolution during cycling. The pristine Ca metal surface inevitably contains electrochemically inert species (e.g., CaO, $Ca(OH)_2$), formed by its reaction with trace water or oxygen. This native passivation layer contributes to a higher interfacial resistance during the initial cycling stages, resulting in a slightly high overpotential. Upon electrochemical cycling, these inert species are gradually replaced by a newly formed SEI with ionically conductive species, facilitating $Ca^{2+}$ transport and reducing overpotential in the later stages of cycling.

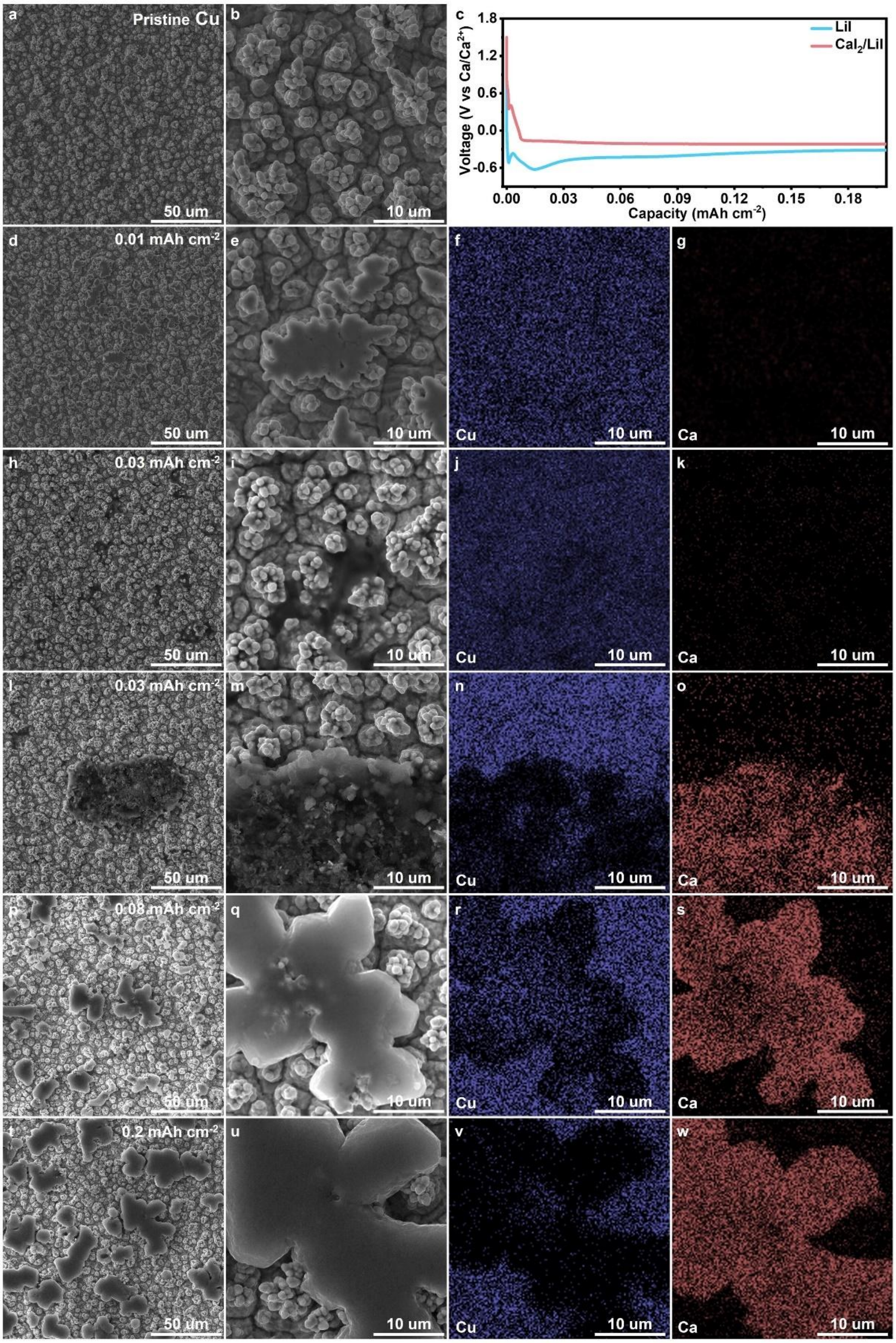


**Figure S3. (a,b)** Scanning electron microscope (SEM) images of pristine Cu current collector. **(c)** Discharge curves of Ca|Cu cells in pure 0.2 M LiI and 0.02 M $CaI_2$/0.2 M

LiI electrolytes. **(d-w)** SEM images and the corresponding energy dispersive spectroscopy (EDS) elemental mappings of Cu current collector with various deposition capacities of Ca|Cu cells in pure 0.2 M LiI electrolytes without $CaI_2$ salt. The electrolyte amount is 80 μL in each cell.

To examine the threshold value of $CaI_2$ concentration for initiating Ca deposition, we collect SEM images and the corresponding EDS mappings of the deposits under different deposition capacities using Ca|Cu cells in pure LiI electrolyte (**Figure S3**). Ca electrode is oxidized to gradually increase $Ca^{2+}$ concentration in the electrolyte. Under a deposition capacity of 0.01 mAh $cm^{-2}$, the partial area of Cu current collector changes into smooth morphologies (**Figure S3d, e**), but the Ca element signal is not detected in the corresponding EDS mapping (**Figure S3g**). This observation indicates $Li^+$ reduction reaction is dominated in the initial deposition process, which is reasonable due to the sole $Li^+$ cation in the original LiI electrolyte. When deposition capacity increases to 0.03 mAh $cm^{-2}$, $Li/Li^+$ redox reaction still exists, as proved by the formed deposits that do not belong to the Ca element signal (**Figure S3h-k**). Meanwhile, Ca element signals are observed in a small area of Cu current collector under 0.03 mAh $cm^{-2}$ (**Figure S3l-o**), suggesting the presence of Ca deposition. Such capacity gives rise to at most 0.007 M $Ca^{2+}$ in the electrolyte released from the Ca electrode. As deposition capacity rises to 0.08 mAh $cm^{-2}$ (equal to ~0.0186 M $Ca^{2+}$ if $Ca^{2+}$ are not consumed), Cu current collector is covered by more deposits that are well overlapped with Ca element signals (**Figure S3p-s**), implying the predominant $Ca/Ca^{2+}$ redox reaction during

electrodeposition. A similar phenomenon is also observed under a higher deposition capacity of 0.2 mAh $cm^{-2}$ (**Figure S3t-w**).

The corresponding discharge curves in pure $CaI_2$ and $CaI_2$/LiI electrolytes agree well with these observations. Note that $Li^+$ cannot be reduced from $CaI_2$/LiI electrolyte, as will be discussed in **Figure 2**. Specifically, Ca|Cu cell experiences a much more negative initial deposition potential in pure LiI electrolyte than in $CaI_2$/LiI counterpart due to the lower redox potential of $Li^+$ than $Ca^{2+}$ (**Figure S3c**). When deposition capacity exceeds 0.015 mAh $cm^{-2}$, the discharge voltage in pure LiI electrolyte continues to increase because of the involvement of Ca deposition. Overall, these results confirm the preferred $Ca^{2+}$ electro-reduction in $CaI_2$/LiI electrolytes, even under an extremely low concentration of $CaI_2$.

In addition, the morphologies of Ca deposition are smooth compared to previous work,[11] primarily stems from the distinct ionic transport properties of the SEIs formed in the two electrolyte systems. Smooth and uniform metal deposition is generally favored when the SEI exhibits high ionic mobility, promoting rapid and homogeneous surface diffusion of $Ca^{2+}$ before deposition. The $Ca(BF_4)_2$-derived SEI, which contains ionic insulating $CaF_2$ species, presents a high energy barrier for $Ca^{2+}$ migration, leading to rough morphology of Ca deposition. In contrast, in our electrolyte system, iodine-rich SEI possesses significantly higher ionic conductivity, facilitating the lateral diffusion of $Ca^{2+}$ across the Ca surface and resulting in the dense and smooth Ca metal morphology.

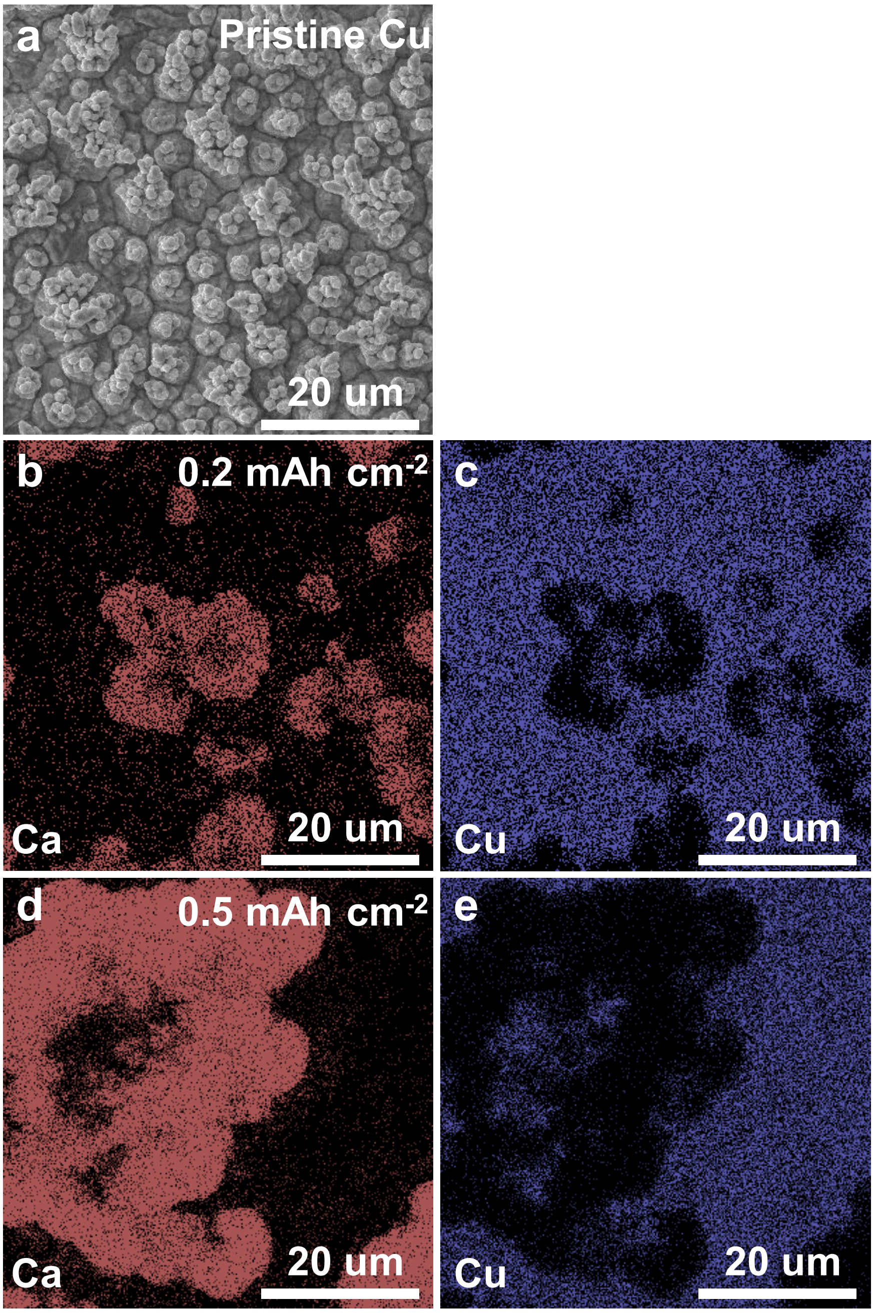


**Figure S4. (a)** SEM image of pristine Cu current collector. EDS elemental mappings of **(b)** Ca and **(c)** Cu of Cu current collector with 0.2 mAh cm$^{-2}$ Ca deposition, and **(d)** Ca and **(e)** Cu of Cu current collector with 0.5 mAh cm$^{-2}$ Ca deposition. The Ca is deposited on Cu current collectors under the $CaI_2$/LiI electrolyte.

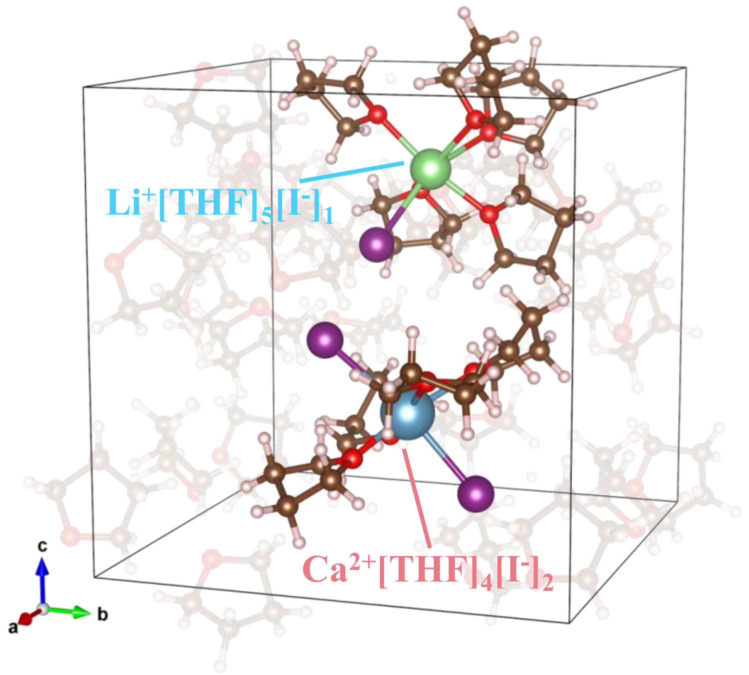


**Figure S5.** Structure of molecular dynamics simulation snapshot used for electronic structure calculation. The standard redox overpotential (calculated in aqueous media) of $Ca/Ca^{2+}$ is 0.17 V higher than that of $Li/Li^{+}$, which is apt to avoid Li metal deposition during the $Ca^{2+}$ electro-reduction process (**Note S1**). Given the possible potential shift caused by the solvents,[22] we compute the electronic structure of $CaI_2$/LiI salt in THF media.

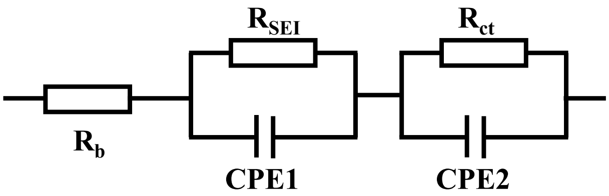


**Figure S6.** Equivalent circuit of EIS Nyquist plots.

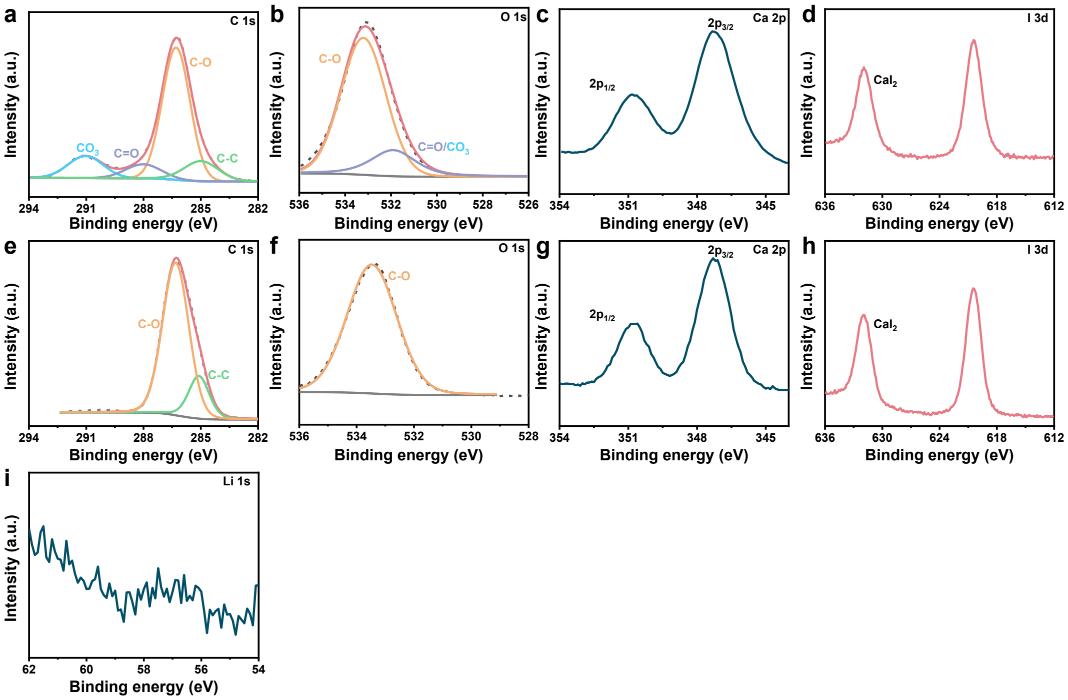


**Figure S7.** XPS spectra of the SEIs formed in **(a-d)** $CaI_2$ and **(e-i)** $CaI_2$/LiI electrolytes before Ar ion sputtering.

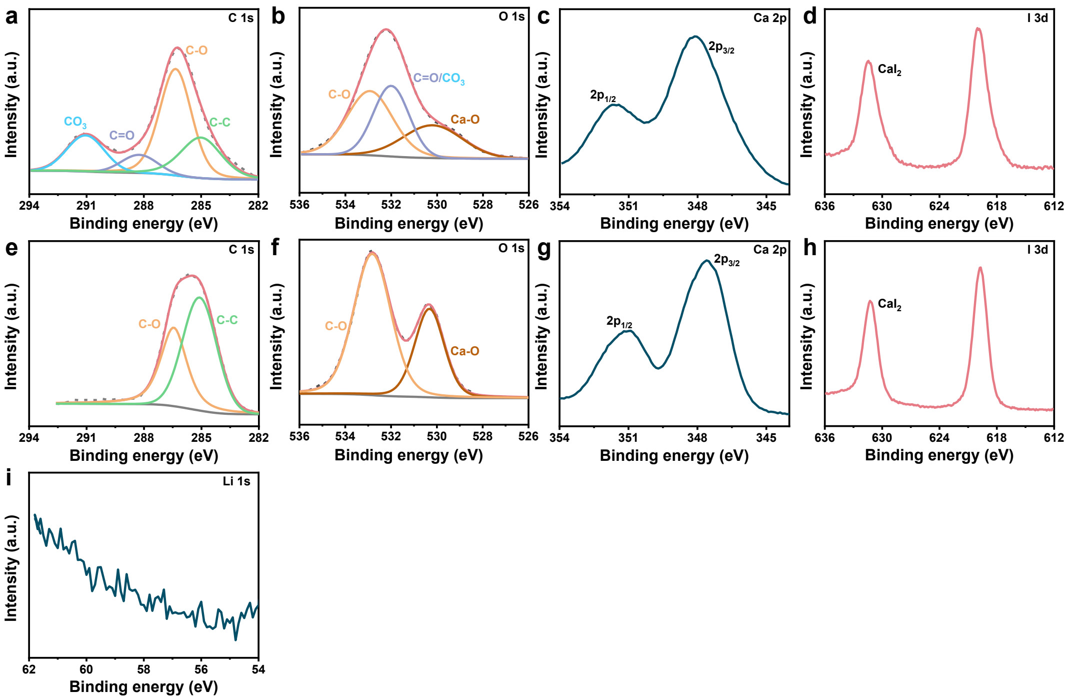


**Figure S8.** XPS spectra of the SEIs formed in **(a-d)** $CaI_2$ and **(e-i)** $CaI_2$/LiI electrolytes after Ar ion sputtering for 150 s.

The absent signal in Li 1s XPS spectra before and after Ar ion sputtering shows that the Li element does not involve in SEI formation under $CaI_2$/LiI electrolyte (**Figure S7** and **8**).

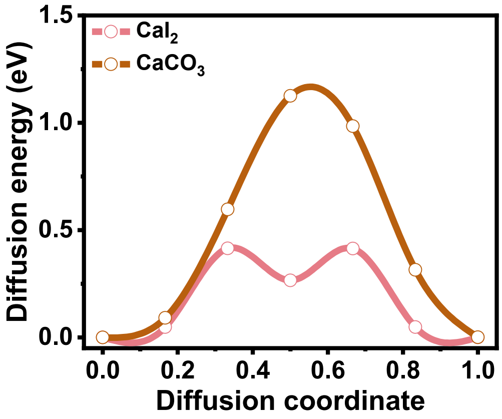


**Figure S9.** Migration barriers in $CaI_2$ and $CaCO_3$, refered to our previous study.[23]

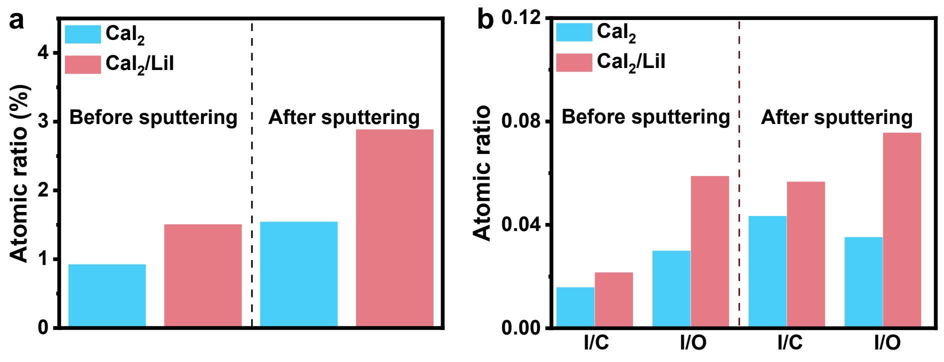


**Figure S10. (a)** I atomic ratio and **(b)** I/O and I/C atomic ratios in SEIs derived from $CaI_2$ and $CaI_2$/LiI electrolytes before and after Ar ion sputtering for 150 s.

As shown in **Figure S10a**, a notable enrichment of $I^-$ within the inner SEI layer is observed in both electrolytes. This is attributed to the preferential adsorption of $I^-$ anions onto the Ca metal surface prior to cycling. The adsorbed $I^-$ is subsequently incorporated into the SEI during its initial formation stage. Besides, at a higher $I^-$ concentration in the electrolyte, the I content in SEI is almost doubled before and after sputtering, demonstrating the increased $CaI_2$ species in SEI under $CaI_2$/LiI electrolyte. I/C and I/O atomic ratios, which can represent the extent of anion/solvent-induced SEIs,[24, 25] are also collected. The increased I/C and I/O atomic ratios are observed in $CaI_2$/LiI electrolyte compared to $CaI_2$ one (**Figure S10b**), suggesting the suppressed solvent decomposition in the former system.

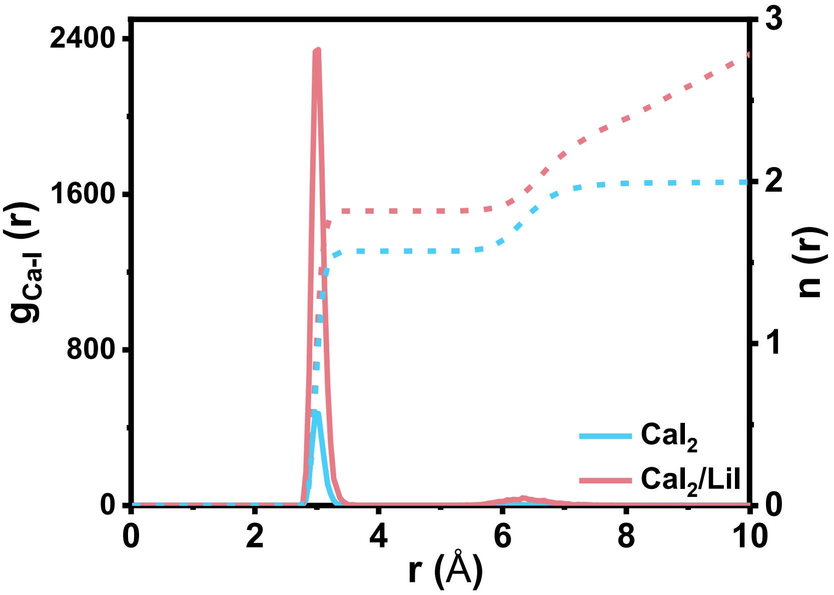


**Figure S11.** Radial distribution function g(r) and integrated coordination number of Ca and I in $CaI_2$ and $CaI_2$/LiI electrolytes.

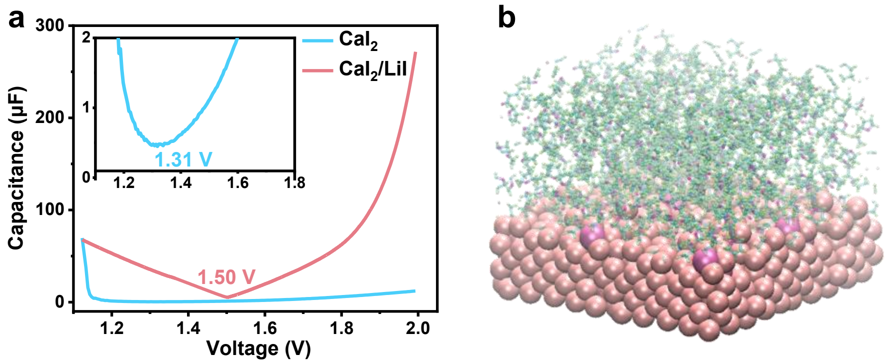


**Figure S12. (a)** The non-Faradaic capacitance-potential in Ca|Cu cells under 1.1-2.0 V (vs. $Ca/Ca^{2+}$). The amplitude is 5 mV with a frequency of 6 Hz. The phase angles of 0 and 90°C are selected. The calculation method of capacitance refers to the previous report.[26] **(b)** Snapshots of MD simulations of the IHP in $CaI_2$-THF electrolyte. The $CaI_2$/LiI electrolyte has a tenfold $I^-$ concentration compared with $CaI_2$ one. Ca and I atoms are shown in opaque pink and purple spheres, respectively.

Alternating current voltammetry (ACV) is carried out to examine the absorption behavior in $CaI_2$ and $CaI_2$/LiI electrolytes. The potential of zero charge (PZC) represents the situation where the minimum capacitance is achieved. The increased absorbed anions will lead to a positive shift of PZC.[27, 28] PZC shifts to a positive potential after adding LiI (**Figure S12a**), suggesting that more I- anions are absorbed in the IHP at a higher $I^-$ concentration. This is further confirmed by the MD simulations where a higher $I^-$ coverage is observed in $CaI_2$/LiI electrolytes (**Figure S12b** and **Figure 3i**).

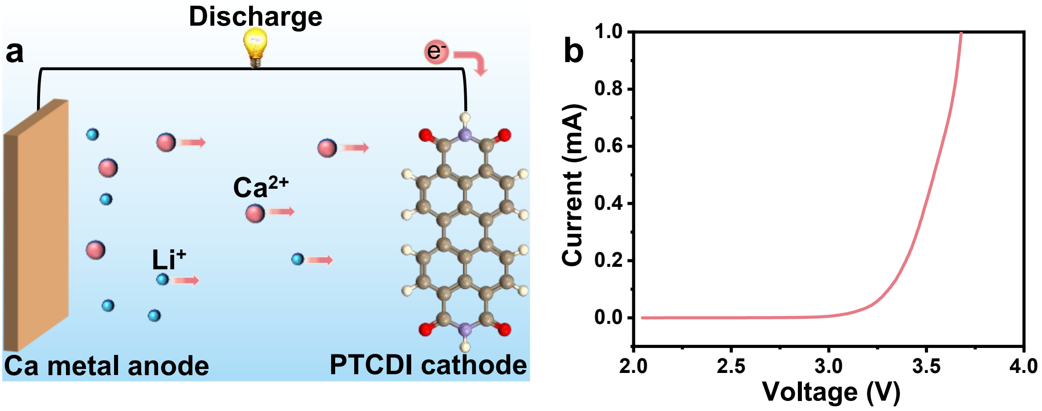


**Figure S13. (a)** Work schematic of Ca|PTCDI full cell during the discharge process. **(b)** Electrochemical window of $CaI_2$/LiI electrolyte using Ca|stainless steel cell at 1 mV $s^{-1}$.

Notably, Ca|PTCDI full cells exhibit better cycling stability at 0°C compared to 25°C (**Figure 4a**). This is mainly because the gradual capacity fading observed at room temperature is primarily attributed to the partial dissolution of the PTCDI active material during cycling—a phenomenon that has been notably documented in previous studies using low salt concentrations.[23] Lowering the operating temperature to 0°C helps suppress this dissolution process, thereby enhancing the cycling stability. This decay can be mitigated through future strategies involving electrolyte engineering and polymer binder optimization.

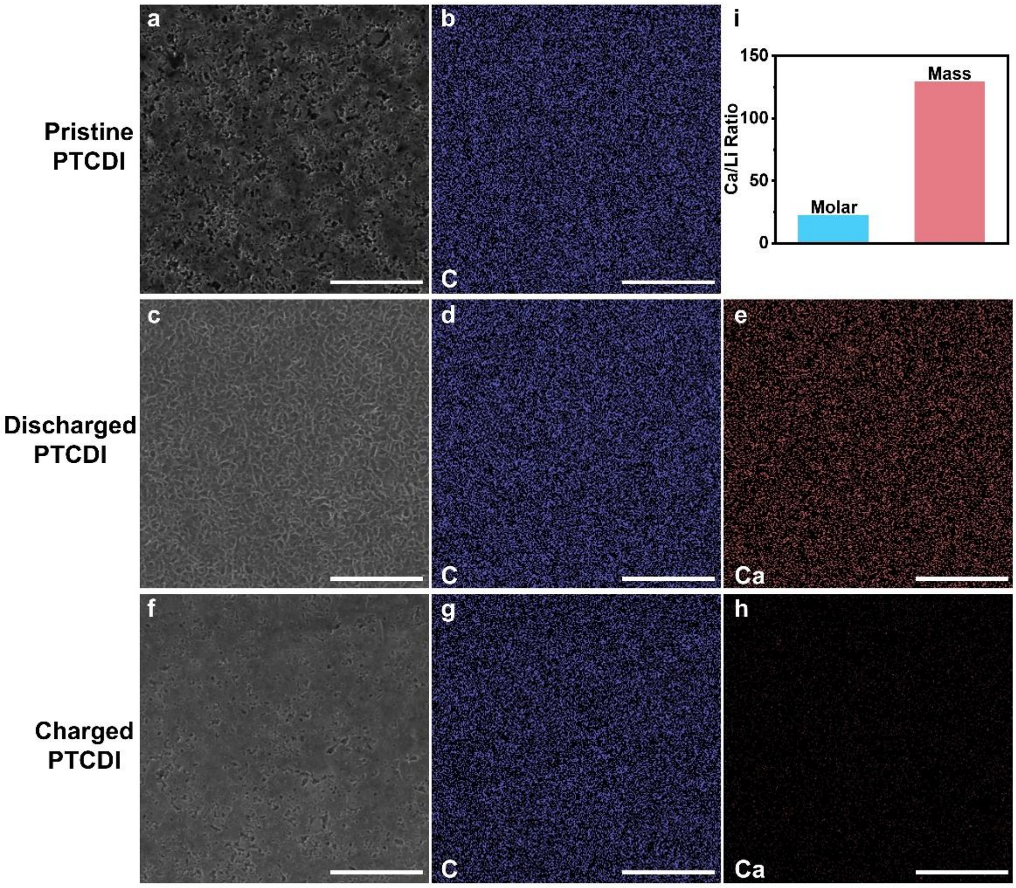


**Figure S14. (a)** SEM image of pristine PTCDI electrode and **(b)** the corresponding EDS elemental mapping of C. **(c)** SEM image of discharged PTCDI electrode and **(d, e)** the corresponding EDS elemental mappings. **(f)** SEM image of charged PTCDI electrode and **(g, h)** the corresponding EDS elemental mappings. Scale bar: 10 μm. **(i)** Ca/Li ratio of discharged PTCDI electrode.

As shown in **Figure S14e**, abundant Ca element signals are detected in discharged PTCDI cathode, which almost disappears in charged PTCDI cathode (**Figure S14h**). The observation indicates extensive $Ca^{2+}$ participation in redox reactions of the cathode reversibly. We collect ICP-MS to determine the Ca/Li ratio in discharged PTCDI cathode. As shown in **Figure S14i**, a high Ca/Li molar ratio of ~22 is observed,

suggesting $Ca^{2+}$ storage is primarily responsible for reversible redox reactions in cathodes.

## References

(1) Kresse, G.; Hafner, J. Ab initio molecular dynamics for open-shell transition metals. *Phys. Rev. B* **1993**, *48* (17), 13115.

(2) Henkelman, G.; Uberuaga, B. P.; Jónsson, H. A climbing image nudged elastic band method for finding saddle points and minimum energy paths. *J. Chem. Phys.* **2000**, *113* (22), 9901-9904.

(3) Grimme, S.; Antony, J.; Ehrlich, S.; Krieg, H. A consistent and accurate ab initio parametrization of density functional dispersion correction (DFT-D) for the 94 elements H-Pu. *J. Chem. Phys.* **2010**, *132* (15), 154104.

(4) Abraham, M. J.; Murtola, T.; Schulz, R.; Páll, S.; Smith, J. C.; Hess, B.; Lindahl, E. GROMACS: High performance molecular simulations through multi-level parallelism from laptops to supercomputers. *SoftwareX* **2015**, *1*, 19-25.

(5) Jorgensen, W. L.; Maxwell, D. S.; Tirado-Rives, J. Development and testing of the OPLS all-atom force field on conformational energetics and properties of organic liquids. *J. Am. Chem. Soc.* **1996**, *118* (45), 11225-11236.

(6) Martínez, L.; Andrade, R.; Birgin, E. G.; Martínez, J. M. PACKMOL: A package for building initial configurations for molecular dynamics simulations. *J. Comput. Chem.* **2009**, *30* (13), 2157-2164.

(7) Hou, Z.; Lu, Z.; Chen, Q.; Zhang, B. Realizing wide-temperature Zn metal anodes through concurrent interface stability regulation and solvation structure modulation. *Energy Storage Mater.* **2021**, *42*, 517-525.

(8) Hou, Z.; Zhou, R.; Min, Z.; Lu, Z.; Zhang, B. Realizing wide-temperature reversible

Ca metal anodes through a $Ca^{2+}$-conducting artificial Layer. *ACS Energy Lett.* **2022**, *8* (1), 274-279.

(9) Hou, Z.; Zhou, R.; Yao, Y.; Min, Z.; Lu, Z.; Zhu, Y.; Tarascon, J. M.; Zhang, B. Correlation between electrolyte chemistry and solid electrolyte interphase for reversible Ca metal anodes. *Angew. Chem. Int. Ed.* **2022**, *61* (50), e202214796.

(10) Thompson, A. P.; Aktulga, H. M.; Berger, R.; Bolintineanu, D. S.; Brown, W. M.; Crozier, P. S.; in't Veld, P. J.; Kohlmeyer, A.; Moore, S. G.; Nguyen, T. D. LAMMPS-a flexible simulation tool for particle-based materials modeling at the atomic, meso, and continuum scales. *Comput. Phys. Commun.* **2022**, *271*, 108171.

(11) Ponrouch, A.; Frontera, C.; Barde, F.; Palacin, M. R. Towards a calcium-based rechargeable battery. *Nat. Mater.* **2016**, *15* (2), 169-172.

(12) Wang, D.; Gao, X.; Chen, Y.; Jin, L.; Kuss, C.; Bruce, P. G. Plating and stripping calcium in an organic electrolyte. *Nat. Mater.* **2018**, *17* (1), 16-20.

(13) Gao, X.; Liu, X.; Mariani, A.; Elia, G. A.; Lechner, M.; Streb, C.; Passerini, S. Alkoxy-functionalized ionic liquid electrolytes: understanding ionic coordination of calcium ion speciation for the rational design of calcium electrolytes. *Energy Environ. Sci.* **2020**, *13* (8), 2559-2569.

(14) Li, Z.; Fuhr, O.; Fichtner, M.; Zhao-Karger, Z. Towards stable and efficient electrolytes for room-temperature rechargeable calcium batteries. *Energy Environ. Sci.* **2019**, *12* (12), 3496-3501.

(15) Shyamsunder, A.; Blanc, L. E.; Assoud, A.; Nazar, L. F. Reversible calcium plating and stripping at room temperature using a borate salt. *ACS Energy Lett.* **2019**, *4* (9),

2271-2276.

(16) Kisu, K.; Kim, S.; Shinohara, T.; Zhao, K.; Zuttel, A.; Orimo, S. I. Monocarborane cluster as a stable fluorine-free calcium battery electrolyte. *Sci. Rep.* **2021**, *11* (1), 7563.

(17) Forero-Saboya, J.; Bodin, C.; Ponrouch, A. A boron-based electrolyte additive for calcium electrodeposition. *Electrochem. Commun.* **2021**, *124*, 106936.

(18) Pavčnik, T.; Forero-Saboya, J. D.; Ponrouch, A.; Robba, A.; Dominko, R.; Bitenc, J. A novel calcium fluorinated alkoxyaluminate salt as a next step towards Ca metal anode rechargeable batteries. *J. Mater. Chem. A* **2023**, *11* (27), 14738-14747.

(19) Leon, N. J.; Xie, X.; Yang, M.; Driscoll, D. M.; Connell, J. G.; Kim, S.; Seguin, T.; Vaughey, J. T.; Balasubramanian, M.; Persson, K. A. Room-temperature calcium plating and stripping using a perfluoroalkoxyaluminate anion electrolyte. *J. Phys. Chem. C* **2022**, *126* (32), 13579-13584.

(20) Aurbach, D.; Skaletsky, R.; Gofer, Y. The electrochemical-behavior of calcium electrodes in a few organic electrolytes. *J. Electrochem. Soc.* **1991**, *138* (12), 3536-3545.

(21) Staniewicz, R. J. A study of the calcium-thionyl chloride electrochemical system. *J. Electrochem. Soc.* **1980**, *127* (4), 782.

(22) Marcus, Y. Thermodynamic functions of transfer of single ions from water to nonaqueous and mixed solvents: Part 3-Standard potentials of selected electrodes. *Pure Appl. Chem.* **1985**, *57* (8), 1129-1132.

(23) Hou, Z.; Zhou, R.; Liu, K.; Zhu, J.; Zhang, B. A $CaI_2$-based electrolyte enabled by borate ester anion receptors for reversible Ca-organic and Ca-Se batteries. *Angew.*

*Chem. Int. Ed.* **2025**, *64* (1), e202413416.

(24) Sayavong, P.; Zhang, W.; Oyakhire, S. T.; Boyle, D. T.; Chen, Y.; Kim, S. C.; Vilá, R. A.; Holmes, S. E.; Kim, M. S.; Bent, S. F. Dissolution of the solid electrolyte interphase and its effects on lithium metal anode cyclability. *J. Am. Chem. Soc.* **2023**, *145*, 12342−12350.

(25) Oyakhire, S. T.; Zhang, W.; Yu, Z.; Holmes, S. E.; Sayavong, P.; Kim, S. C.; Boyle, D. T.; Kim, M. S.; Zhang, Z.; Cui, Y. Correlating the formation protocols of solid electrolyte interphases with practical performance metrics in lithium metal batteries. *ACS Energy Lett.* **2023**, *8*, 869-877.

(26) Herzog, G.; Moujahid, W.; Strutwolf, J.; Arrigan, D. W. Interactions of proteins with small ionised molecules: electrochemical adsorption and facilitated ion transfer voltammetry of haemoglobin at the liquid| liquid interface. *Analyst* **2009**, *134* (8), 1608-1613.

(27) Xiao, D.; Li, Q.; Luo, D.; Gao, R.; Li, Z.; Feng, M.; Or, T.; Shui, L.; Zhou, G.; Wang, X. Establishing the preferential adsorption of anion-dominated solvation structures in the electrolytes for high-energy-density lithium metal batteries. *Adv. Funct. Mater.* **2021**, *31* (30), 2011109.

(28) Yang, H.; Li, Y.; Zhou, X.; Ma, X.; Duan, D.; Liu, S. Effect of specifically-adsorbed polysulfides on the electron transfer kinetics of sodium metal anodes. *J. Energy Chem.* **2022**, *74*, 26-33.
